# Advances in the Insulin–Heart Axis: Current Therapies and Future Directions

**DOI:** 10.3390/ijms251810173

**Published:** 2024-09-22

**Authors:** Alfredo Caturano, Erica Vetrano, Raffaele Galiero, Celestino Sardu, Luca Rinaldi, Vincenzo Russo, Marcellino Monda, Raffaele Marfella, Ferdinando Carlo Sasso

**Affiliations:** 1 Department of Advanced Medical and Surgical Sciences, University of Campania Luigi Vanvitelli, 80138 Naples, Italy; alfredo.caturano@unicampania.it (A.C.); erica.vetrano@unicampania.it (E.V.); raffaele.galiero@unicampania.it (R.G.); celestino.sardu@unicampania.it (C.S.); raffaele.marfella@unicampania.it (R.M.); 2 Department of Experimental Medicine, University of Campania Luigi Vanvitelli, 80138 Naples, Italy; marcellino.monda@unicampania.it; 3 Department of Medicine and Health Sciences “Vincenzo Tiberio”, University of Molise, 86100 Campobasso, Italy; 4 Department of Biology, College of Science and Technology, Sbarro Institute for Cancer Research and Molecular Medicine, Temple University, Philadelphia, PA 19122, USA; v.p.russo@libero.it; 5 Division of Cardiology, Department of Medical Translational Sciences, University of Campania Luigi Vanvitelli, 80138 Naples, Italy

**Keywords:** insulin–heart axis, cardiovascular pharmacotherapy, heart failure management, insulin sensitivity, insulin resistance

## Abstract

The insulin–heart axis plays a pivotal role in the pathophysiology of cardiovascular disease (CVD) in insulin-resistant states, including type 2 diabetes mellitus. Insulin resistance disrupts glucose and lipid metabolism, leading to systemic inflammation, oxidative stress, and atherogenesis, which contribute to heart failure (HF) and other CVDs. This review was conducted by systematically searching PubMed, Scopus, and Web of Science databases for peer-reviewed studies published in the past decade, focusing on therapeutic interventions targeting the insulin–heart axis. Studies were selected based on their relevance to insulin resistance, cardiovascular outcomes, and the efficacy of pharmacologic treatments. Key findings from the review highlight the efficacy of lifestyle modifications, such as dietary changes and physical activity, which remain the cornerstone of managing insulin resistance and improving cardiovascular outcomes. Moreover, pharmacologic interventions, such as metformin, sodium–glucose cotransporter 2 inhibitors, glucagon-like peptide-1 receptor agonists, and dipeptidyl peptidase-4 inhibitors, have shown efficacy in reducing cardiovascular risk by addressing metabolic dysfunction, reducing inflammation, and improving endothelial function. Furthermore, emerging treatments, such as angiotensin receptor–neprilysin inhibitors, and mechanical interventions like ventricular assist devices offer new avenues for managing HF in insulin-resistant patients. The potential of these therapies to improve left ventricular ejection fraction and reverse pathological cardiac remodeling highlights the importance of early intervention. However, challenges remain in optimizing treatment regimens and understanding the long-term cardiovascular effects of these agents. Future research should focus on personalized approaches that integrate lifestyle and pharmacologic therapies to effectively target the insulin–heart axis and mitigate the burden of cardiovascular complications in insulin-resistant populations.

## 1. Introduction

Insulin resistance is a condition in which insulin is unable to exert its normal biological activity of facilitating the entry of glucose into insulin-sensitive tissues to be used as the primary energy substrate [1]. This metabolic dysregulation primarily affects four key organs, once termed “the deadly quartet”: adipose tissue, muscle, liver, and the endocrine pancreas. Each of these organs plays distinct yet interrelated roles in the development and maintenance of insulin resistance and its progression to type 2 diabetes mellitus (T2DM) [2]. Interestingly, in vivo studies have shown that insulin leads to a substantial increase in left ventricular ejection fraction (LVEF) following submaximal exercise. However, this increase is notably reduced in individuals with insulin resistance, including those with T2DM [3] and obese non-diabetic individuals [4]. Persistent hyperglycemia results in significant metabolic disturbances across these organs, causing alterations in their metabolic pathways. These disturbances manifest in a variety of complications, including impaired adipose tissue function, obesity, inflammation, dyslipidemia, and increased production of reactive oxidative species (ROS). These factors collectively contribute to atherosclerosis, endothelial dysfunction, and hypertension, all of which are major risk factors for cardiovascular disease (CVD) [1,5,6,7]. Visceral obesity is one of the clinical manifestations most typically associated with insulin resistance and cardiometabolic alterations [8,9]. Visceral adipose tissue (VAT) is a major source of proinflammatory adipokines, such as tumor necrosis factor-alpha (TNF-α), interleukin 1-beta (IL-1β), and interleukin 6 (IL-6), as well as chemotactic factors like monocyte chemoattractant protein-1 (MCP-1). These inflammatory mediators induce molecular changes in adipocytes, diminishing their insulin sensitivity. Consequently, glucose uptake by cells is reduced, and while triglycerides accumulate in adipocytes, fat deposition shifts to other organs such as the pancreas, liver, kidneys, vessels, skeletal muscle, heart, and epicardial adipose tissue (EAT). This redistribution adversely affects insulin sensitivity and organ function [10,11,12]. In EAT, fat accumulation leads to structural changes in the heart, including myocardial fibrosis, cardiac steatosis, and ventricular hypertrophy, which contribute to both contractile and diastolic dysfunction [13,14]. Additionally, VAT’s altered hormonal activity causes the liver to capture increased amounts of fatty acids released from adipose tissue, resulting in hepatic fat accumulation. This, in turn, triggers an inflammatory response, oxidative stress, and disrupted autophagy, which collectively contribute to the progression of metabolic-dysfunction-associated steatotic liver disease (MASLD) [15,16]. The interaction between VAT and the liver, with VAT secreting most inflammatory and extracellular matrix (ECM) proteins, plays a crucial role in MASLD’s pathogenesis [17,18,19].

Insulin resistance also affects lipid metabolism, leading to an atherogenic lipid profile characterized by hypertriglyceridemia, elevated levels of very low-density lipoprotein (VLDL), decreased high-density lipoprotein (HDL), and increased formation of small dense low-density lipoprotein (sdLDL) [20]. Insulin regulates VLDL synthesis through the PI3K pathway by promoting the degradation of apoprotein B (apoB), thus inhibiting VLDL assembly and secretion. In insulin resistance, this mechanism is impaired, resulting in increased production of triglyceride-rich VLDL and reduced lipoprotein clearance [21]. The formation of sdLDL is influenced by the dysfunctional cholesteryl ester transfer protein (CEPT) and hepatic lipase, leading to triglyceride accumulation in LDL and HDL, transforming LDL into sdLDL, and increasing HDL catabolism [22,23]. This lipid profile is highly atherogenic as sdLDLs penetrate the vascular wall more readily, have a longer half-life, are more prone to oxidation, and have reduced receptor affinity [24].

Insulin resistance is also linked to hypertension, with several alterations synergistically impairing endothelial function and disrupting the balance between vasoconstrictors and vasodilators [13,25,26]. Hypertriglyceridemia activates the renin–angiotensin–aldosterone system (RAAS), which, in concert with insulin, stimulates the MAPK pathway, leading to increased proliferation and contractility of vascular myocytes and endothelial inflammation [13,27]. RAAS further disrupts insulin signaling by promoting phosphorylation of IRS-1 and IRS-2, activating mTOR-S6K1, and impeding insulin signal transduction through the PI3K pathway. This results in decreased production of endothelial nitric oxide (eNO), a potent vasodilator essential for vascular protection [28]. In insulin-resistant individuals, eNO production is reduced due to impaired PI3K signaling, while compensatory hyperinsulinemia activates the MAPK pathway, leading to myocyte proliferation, vasoconstriction, fluid retention, and elevated blood pressure [29]. Moreover, hyperactivation of the sympathetic nervous system induces vascular changes, including myocyte hypertrophy, apoptosis, interstitial fibrosis, and reduced contractile function [30]. Aldosterone and insulin both enhance the activity of serine/threonine protein kinase SGK-1, which regulates vascular and renal sodium channel activity. Mutations in SGK-1 are associated with hypertension, insulin resistance, and obesity. Increased sodium flux in endothelial cells contributes to cytoskeletal remodeling, reduced NO bioavailability, and vascular stiffening [31]. Hyperglycemia negatively impacts coronary circulation and cardiomyocyte membrane stability by disrupting Ca^2+^ homeostasis and reducing cardiomyocyte survival [32]. Insulin resistance impairs coronary microcirculation and reduces coronary flow reserve, potentially damaging vasa vasorum and microcirculation, leading to vasospasm [33]. Studies indicate that insulin-resistant environments are marked by factors like TNF-α and vascular endothelial growth factor (VEGF) that hinder vascular maturation, resulting in weaker, smaller vessels prone to rupture, thrombosis, and cardiovascular events [34]. Additionally, exosomes from insulin-resistant adipocytes can exacerbate insulin resistance, promote vasa vasorum angiogenesis, and increase plaque burden and vulnerability in ApoE^−/−^ diabetic mice [35]. The molecular alterations associated with insulin resistance illustrate its profound impact on cardiovascular health [36]. Insulin signaling is crucial for heart growth, survival, substrate utilization, and mitochondrial metabolism. Disruptions in insulin signaling contribute to pathological ventricular remodeling and heart failure (HF). Hyperinsulinemia, through sodium retention, increases myocardial mass and induces subclinical myocardial dysfunction. It also activates the sympathetic nervous system, impairing cardiac function and promoting ventricular hypertrophy. This remodeling leads to increased left ventricular mass, myocardial oxygen demand, and reduced diastolic pressure, further impairing coronary blood flow [37].

The condition of HF itself can induce and exacerbate insulin resistance, creating a vicious cycle [38,39]. Norepinephrine release due to reduced arterial filling in HF [40] impairs insulin sensitivity and glucose tolerance, aggravating insulin resistance [41]. This interplay results in pathological left ventricular remodeling characteristics of diabetic cardiomyopathy, which is prevalent in T2DM and strongly predicts adverse cardiovascular outcomes, particularly heart failure with preserved ejection fraction (HFpEF) [42,43]. Elevated fat deposition in the myocardium can increase ventricular mass and alter cardiac structure and function through toxic lipid accumulation [22,44,45]. Studies have shown that hyperinsulinemia and waist-to-hip ratio are linked to left ventricular concentric remodeling, indicating that addressing insulin resistance may help reverse such remodeling and improve HF prognosis [46,47].

**Figure 1:**
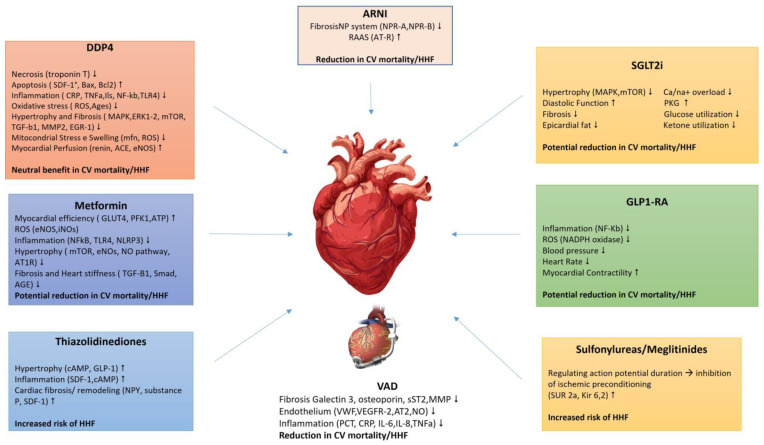
Cardiovascular Effects of Diabetes Medications and Heart Failure Therapies.

Diabetes significantly increases the risk of morbidity and mortality in patients with heart failure, whether with reduced (HFrEF), mid-range (HFmrEF), or HFpEF [48]. The Phosphodiesterase-5 Inhibition to Improve Clinical Status and Exercise Capacity in Heart Failure with Preserved Ejection Fraction study (RELAX) (NCT00763867) highlighted that patients with HFpEF and diabetes have elevated levels of endothelin-1 (ET-1), inflammatory markers, and profibrotic indicators, correlating with worse left ventricular diastolic function, increased left ventricular mass, and reduced exercise tolerance compared to non-diabetic HF patients [49,50]. Echocardiographic assessments reveal that patients with HF and T2DM exhibit notably poorer left ventricular diastolic function, as indicated by a higher E/e’ ratio, increased left ventricular mass, and diminished exercise tolerance compared to HF patients without diabetes [51]. Insulin resistance in HF patients is closely tied to prognosis, with impaired left ventricular ejection fraction (LVEF) and difficulty improving LVEF observed in those with HF and insulin resistance [52,53]. Insulin resistance in HF patients is closely linked to prognosis. Those with HF and insulin resistance face greater challenges in enhancing their LVEF, suggesting that insulin resistance is independently associated with the inability to improve LVEF in individuals with HF with HFrEF [54]. Over the past decade, advancements in HF treatments have enabled many patients with HFrEF and those with HFmrEF to achieve near-normal levels of LVEF [55]. Addressing insulin resistance and its cardiovascular implications requires a comprehensive approach, including lifestyle modifications and pharmacological treatments [56,57]. Given the intricate relationship between insulin signaling and cardiac function, the insulin–heart axis represents a critical therapeutic target. This manuscript aims to review the current pharmacological agents that target this axis, examining their mechanisms of action, clinical efficacy (Figure 1), and cardiovascular benefits to optimize treatment strategies for patients with insulin resistance and related cardiovascular disorders.

This figure summarizes the cardiovascular effects of various diabetes medications and heart failure therapies. Each drug class or intervention is linked to specific mechanisms and outcomes related to heart function. Notably, SGLT2 inhibitors, GLP-1 receptor agonists, and metformin show potential reductions in cardiovascular mortality and heart failure hospitalization (HHF) through mechanisms such as reducing fibrosis, inflammation, and oxidative stress. ARNI and ventricular assist devices (VADs) provide beneficial effects by modulating the RAAS, fibrosis, and inflammation, also reducing cardiovascular mortality and HHF. In contrast, thiazolidinediones and sulfonylureas/meglitinides are associated with an increased risk of HHF due to their influence on cardiac hypertrophy, fibrosis, and action potential regulation. DPP4 inhibitors demonstrate a neutral benefit in terms of cardiovascular mortality and HHF.

## 2. Lifestyle Modification

The gold standard for the treatment of insulin resistance involves a balanced, low-carbohydrate, low-calorie diet in obese and overweight individuals, along with moderate and consistent physical activity when possible [58]. The American Heart Association (AHA) recognizes that diet and lifestyle are important modifiable factors influencing the risk of coronary heart disease and are key components in preventing cardiovascular risk [59]. The Mediterranean diet, which includes a variety of fruits, vegetables, whole grains, and healthy fats, has been shown to improve insulin sensitivity and reduce the risk of cardiovascular disease. Limiting the intake of refined carbohydrates and added sugars is essential because these dietary elements lead to increased blood sugar levels after meals and insulin resistance [60]. Among various diets, very low-calorie ketogenic diets (VLCKDs) deserve special mention due to their successful use in obese or overweight individuals with prediabetes or diabetes, which are low-calorie diets (between 600 and 800 Kcal/day) with a low carbohydrate content (20 to 60 g/day). According to international guidelines, VLCKDs can be used continuously for 12 weeks under medical supervision [61]. The drastic reduction in carbohydrate intake results in increased fat catabolism, with an imbalance between the production of pyruvate, oxaloacetate, and acetyl-coenzyme A (acetyl-CoA), leading to the formation of ketone bodies. The heart, which has a high energy demand, primarily relies on mitochondrial oxidation of fatty acids and glucose. In HF, reduced mitochondrial oxidative metabolism and glucose oxidation make the heart energy-deficient [62]. Ketone bodies can be rapidly oxidized by heart muscle, providing an additional energy source for the compromised heart [63]. On the other hand, a high-fat meal is associated with an increased production of TNF-α, a proinflammatory cytokine, which can contribute to endothelial dysfunction. Elevated TNF-α levels are commonly observed in individuals with metabolic syndrome, where they play a key role in promoting inflammation and insulin resistance. The acute rise in TNF-α following a high-fat meal exacerbates this inflammatory response, leading to impaired endothelial function, which is a critical factor in the development of cardiovascular diseases [64].

Consistent exercise is crucial for managing insulin resistance, as it positively impacts glucose metabolism and cardiovascular well-being [65]. Both aerobic exercise and resistance training improve insulin sensitivity [66]. For optimal metabolic health, it is recommended to participate in at least 150 min of moderate aerobic activity each week and engage in strength training activities at least twice a week. Physical activity stimulates the movement of glucose transporters to the cell membrane, facilitating glucose absorption by skeletal muscle cells [67,68]. Additionally, exercise improves mitochondrial function, reducing levels of oxidative stress and inflammation related to insulin resistance [69]. Weight loss, especially in obese individuals, significantly impacts insulin sensitivity. Even a slight weight reduction improves the body’s response to insulin and reduces the likelihood of cardiovascular events [70]. In cases of severe obesity, bariatric surgery not only results in significant weight loss but also markedly improves insulin sensitivity and metabolic parameters. The interaction of lifestyle factors underscores the importance of a comprehensive strategy to address insulin resistance. The combination of dietary adjustments, physical activity, and weight control produces a synergistic impact, effectively managing various aspects of insulin resistance [62,71,72]. However, maintaining a healthy lifestyle is consistently carried out by only a small percentage of patients, while the majority eventually revert to harmful habits. To improve adherence, patients need to be well informed and regularly reminded of the risks associated with non-adherence [73,74].

## 3. Metformin

Metformin, a historical oral antidiabetic drug belonging to the biguanide class, is used as a first-line treatment for T2DM [75]. Metformin’s ability to reduce diabetes-related cardiovascular risk derives from its direct effects on the endothelium, independent of improvements in metabolic disorders (e.g., insulin resistance and hyperglycemia) and commonly associated risk factors (e.g., dyslipidemia and hypertension) [76,77,78,79]. Metformin reduces glucose production in the liver by activating adenosine monophosphate-activated protein kinase (AMPK), a critical regulator of cellular energy homeostasis that coordinates enzymes involved in carbohydrate and fat metabolism to enable ATP storage and synthesis [80]. Evidence suggests that AMPK’s role extends beyond energy metabolism regulation, as the enzyme can influence a wide range of cellular functions, representing various pleiotropic actions of metformin [81]. For example, AMPK stimulates the production of eNOS, supporting a protective role on the endothelium, as demonstrated in a study on obese rats [82]. Metformin, by activating AMPK, can also induce regression of myocardial hypertrophy [83,84]. This drug improves insulin sensitivity by increasing insulin receptor tyrosine kinase activity, promoting glycogen synthesis, and enhancing glucose transporter 4 (GLUT4) recruitment and activity [85]. Metformin appears to have numerous favorable effects in HF patients, helping to reduce the incidence of heart disease. In a mouse model of HF, metformin treatment attenuated myocardial fibrosis by inhibiting the TGFβ1-Smad3 signaling pathway [86]. Studies have shown that metformin provides several benefits through its effects on macrophages. In human macrophages, metformin treatment selectively inhibited the differentiation of human monocytes into proinflammatory M1 macrophages [87]. Additionally, LPS stimulated M2 macrophages to produce ROS that is detrimental to surrounding tissues, a process inhibited by metformin. Metformin reduces oxidative stress and inflammatory responses by inhibiting the differentiation of human monocytes into M1 macrophages and limiting macrophage ROS production through AMPK activation [87]. Metformin may also exert anti-inflammatory effects by modulating the AMPK/mTOR signaling pathway to inhibit NLRP3 inflammasome activation and promote macrophage polarization toward the M2 phenotype [88]. These results suggest that metformin acts as a cardioprotective and anti-inflammatory agent by stimulating AMPK/autophagy and thereby inhibiting the NLRP3 inflammasome, closely associated with macrophages in diabetic cardiomyopathy. Thus, through improved lipid profile, reduced inflammatory status, oxidative stress, and hyperinsulinemia, metformin can significantly regress atherosclerotic lesions and enhance endothelial function by limiting endothelial production of adhesion molecules, monocyte infiltration, and foam cell formation [89]. Metformin has also been shown to improve the dynamics of microvascular circulation [90,91]. Often overlooked are metformin’s effects on hemostasis and thrombolysis, particularly in decreasing platelet aggregation, altering fibrin polymerization, and reducing plasminogen activator inhibitor-1 (PAI-1) [92]. Despite this catalog of beneficial effects on the vascular system, most studies with metformin have noted little effect on blood pressure, beyond small reductions consistent with decreased adiposity, and also an antitumor effect [93,94]. Several studies continue to examine the cardiovascular effects of metformin, and several prospective studies with cardiovascular endpoints are ongoing, notably SGLT2 Inhibitor or Metformin as Standard Treatment of Early Stage Type 2 Diabetes (SMARTEST) (NCT03982381) [95,96], Metformin and Prevention of Cardiovascular Events in Patients With Acute Myocardial Infarction and Prediabetes (MIMET) (NCT05182970) [97,98], DANHEART (H-HeFT and Met-HeFT) (NCT03514108) [99,100], and Investigation of Metformin in Pre-Diabetes on Atherosclerotic Cardiovascular OuTcomes (VA-IMPACT) (NCT02915198) [101]. However, it is unlikely that very large prospective randomized cardiovascular outcomes studies will be conducted with this drug for comparison with each new antihyperglycemic agent; most patients recruited in these trials are taking new antihyperglycemic agents in addition to metformin, so the new agents are largely tested as adjunctive therapies to metformin.

## 4. SGLT2 Inhibitors

The SGLT2 inhibitors approved by the United States Food and Drug Administration (FDA) and/or similar regulatory agencies in the European Union and other countries include empagliflozin, dapagliflozin, canagliflozin, ertugliflozin, ipragliflozin, tofogliflozin, luseogliflozin, and remogliflozin [102]. They are a new class of oral antihyperglycemic agents that block renal glucose reabsorption, reduce body weight, improve visceral adiposity and blood pressure, and normalize lipid profile and serum uric acid levels [103,104,105]. By promoting glycosuria, these agents reduce blood glucose levels, leading to improved insulin sensitivity. This reduction in glucose reabsorption results in decreased plasma glucose levels and a consequent decrease in insulin demand, which helps alleviate the burden on pancreatic beta-cells and improve overall insulin action [106,107]. Moreover, SGLT2 inhibitors have been linked to improved lipid profiles and a reduction in blood pressure, which further supports their role in mitigating insulin resistance. The combined effect of these metabolic benefits translates into better glucose control and a reduced risk of cardiovascular events, which are commonly seen in patients with insulin resistance and T2DM [102,108]. The significant cardiac and renal protective characteristics of SGLT2 inhibitors have resulted in their widespread use as monotherapy or adjunctive therapy with metformin, sulfonylureas, glucagon-like peptide-1 receptor agonists, thiazolidinediones, and insulin for the management of T2DM, as well as HF with reduced and preserved ejection fraction, as suggested by recent guidelines [109,110,111]. The use of SGLT2 inhibitors has been associated with cardiovascular benefits, including reduction of collagen production, suppression of fibroblast activation, and reduction of sympathetic overdrive [112,113,114]. They also increase natriuresis and osmotic diuresis, leading to decreased renin production and reduced filtration rate, intraglomerular hydrostatic pressure, and blood pressure [115]. Additional mechanisms proposed for SGLT2 inhibitors include remodeling of the nephron, relaxation of vascular smooth muscle cells, and weight loss independent of fluid contraction caused by glycosuria [116]. Interestingly, SGLT2 inhibitors play a role in the inflammatory response, which may help reduce unfavorable cardiac remodeling and improve cardiovascular outcomes. For instance, following ischemia–reperfusion injury, SGLT2 knockdown animals showed decreased oxidative stress, resulting in less myocardial necrosis and smaller infarct size. The NADPH oxidase 2 (NOX2) was downregulated, mediating these effects [117]. Thus, SGLT2 inhibitors have been linked to anti-inflammatory actions [118]. Dapagliflozin reduced collagen production after myocardial infarction (MI) in mice by activating anti-inflammatory macrophages and suppressing myofibroblast development [119]. In a dose-dependent manner, empagliflozin inhibited the activation of human fibroblasts by transforming growth factor 1 (TGF1). The empagliflozin group also had a lower level of profibrotic markers (such as collagen type I chain 1 and matrix metallopeptidase 2) than the placebo group [120]. As a result, empagliflozin was found to reduce sympathetic overdrive (i.e., catecholamine levels), a factor in neurohormonal activation and a key indicator of adverse cardiac remodeling [121,122,123]. Administration of empagliflozin for 10 weeks was associated with a reduction in atherosclerotic lesion formation in the aorta in an apolipoprotein E (APOE) mutant mouse model fed a high-fat diet. Furthermore, while SGLT1 was expressed in all aortic samples, SGLT2 was found in only a small number of them [124]. Empagliflozin has also been demonstrated to reduce inflammation in the kidney and substantially reduce renal production of proinflammatory cytokines and chemokines (including TNF-α), urinary indicators of renal inflammation (such as IL-6), and apoptosis in a diabetes-induced rat model [125]. In this study, empagliflozin was also associated with reduced expression of profibrotic genes such as TGF, collagen type IV, and fibronectin [126]. In numerous inflammatory models, canagliflozin has been found to possess anti-inflammatory properties. By decreasing tumor necrosis factor receptor 1 (TNFR1) and IL-6, canagliflozin aids in reversing molecular processes associated with inflammation, extracellular matrix turnover, and fibrosis [127]. The SGLT2 inhibitors showed a higher rate of osmotic diuresis and natriuresis. Increased natriuresis and osmotic diuresis are associated with decreased renin production by the juxtaglomerular apparatus, vasoconstriction of the afferent arteriole, and vasodilation of the efferent arteriole at the glomeruli, subsequently leading to a decrease in filtration rate and intraglomerular hydrostatic pressure [128]. Patients treated with SGLT2 inhibitors showed a change in systolic/diastolic blood pressure (BP) of −3.8/−1.5 mmHg from baseline compared to placebo-treated patients [129]. The use of SGLT2 inhibitors lowered glycated hemoglobin by 0.46% compared to placebo, and this reduction in blood glucose was linked to a lower risk of all fatal and non-fatal events except stroke [130]. The fact that the increase in urinary volume returns to pretreatment levels after three months and the reduction in blood pressure lasts almost four years suggests that osmotic diuresis is not the main mechanism causing the reduction in blood pressure. Other proposed mechanisms include nephron remodeling that reduces arterial stiffness, relaxation of vascular smooth muscle cells due to a negative sodium balance, reduced sympathetic activity, and weight loss independent of fluid contraction caused by glycosuria [105]. Finally, these drugs also increase the production of ketone bodies, which serve as an alternative energy source for the heart [131]. Ketone bodies are more efficient than glucose in generating ATP, which may be particularly beneficial in the context of heart failure [132]. In addition to their energy efficiency, ketone bodies have been shown to improve insulin sensitivity in cardiac tissue [133]. This enhanced insulin sensitivity may reduce insulin resistance in the heart, thereby improving cardiac function and offering potential cardioprotective effects. This dual action of SGLT2 inhibitors—reducing glucose levels and promoting ketone body production—may be a key factor in their beneficial effects on heart health [134].

## 5. GLP-1 Receptor Agonists

Glucagon-like peptide-1 (GLP-1) is an incretin hormone whose action is mediated by a specific receptor (GLP-1 receptor, GLP-1R) located in the intestine (distal ileum and colon), pancreatic alpha and beta-cells, and the central nervous system (CNS) [135]. Although at lower levels, it is also expressed in the heart, lungs, kidneys, blood vessels, and peripheral nervous system [135]. The primary effect of GLP-1 is the enhancement of insulin secretion from beta-cells in response to hyperglycemia and the inhibition of glucagon release from alpha-cells [136]. Additionally, GLP-1 slows gastric emptying and gastrointestinal motility to reduce glucose absorption, helping to control postprandial glucose and triglyceride levels. The use of GLP-1 also induces satiety through direct stimulation of the CNS [137,138]. Glucagon-like peptide-1 receptor agonists (GLP-1 RAs) are utilized in the treatment of T2DM and obesity, with agents such as semaglutide, albiglutide, dulaglutide, exenatide, liraglutide, and lixisenatide currently in use [139]. Although the primary focus of GLP-1 RAs has been on glucose control, these agents have demonstrated cardiovascular benefits, which are particularly relevant in the management of the insulin–heart axis. For instance, GLP-1 RAs have been shown to improve endothelial function, reduce oxidative stress, and modulate inflammation, all of which are crucial for cardiovascular protection in insulin-resistant individuals [140]. Liraglutide, semaglutide, and albiglutide have been associated with a reduced risk of major adverse cardiac events, while exenatide and lixisenatide exhibit neutral effects on these outcomes [141]. Moreover, GLP-1 RAs have shown promise in addressing HF, particularly in reducing the risk of developing HF in patients with diabetes. However, their effects in individuals with established HF still need to be clarified [142]. A recent meta-analysis involving 54,092 participants from seven randomized controlled trials, which included individuals with T2DM and a history of HF (16%), suggested that GLP-1 RAs might help prevent new-onset HF in the diabetic population. However, in patients with pre-existing HF, these drugs did not significantly reduce the risk of HF exacerbation or mortality [143]. A similar meta-analysis, which included 68,653 patients across 10 trials, found that while GLP-1 receptor agonists did not reduce the risk of hospitalization for HF, they may offer potential benefits in patients without a prior history of HF. Additionally, these agents were shown to be effective in reducing ischemic events, regardless of whether patients had HF [144]. Liraglutide, semaglutide, and albiglutide have been shown to reduce the risk of major adverse cardiac events, whereas exenatide and lixisenatide exhibit neutral effects [145]. Another meta-analysis suggested that all GLP-1 receptor agonists are capable of reducing cardiovascular incidents, cardiovascular mortality, and all-cause mortality to varying degrees, with no significant adverse effects, allowing for personalized drug regimens [146]. Despite these mixed results, the potential of GLP-1 RAs to modulate key pathways involved in HF, such as inflammation, endothelial function, and atherosclerosis, positions them as valuable tools in the broader management of cardiovascular risk in insulin-resistant patients [147,148,149]. Furthermore, GLP-1 RAs have been found to enhance nitric oxide (NO) production and activate several kinases in cardiomyocytes, including Akt-1, PI-3K, and MAPK. These actions potentiate glucose uptake and provide additional cardioprotection against ischemia, thereby offering a dual benefit of glucose control and cardiovascular protection. Additionally, NO production by GLP-1 RAs in endothelial cells promotes vasodilation, further supporting cardiovascular health [150].

In recent developments, tirzepatide, a dual gastric inhibitory peptide (GIP) and GLP-1 receptor agonist, has demonstrated favorable modulation of metabolites related to insulin resistance and future T2DM risk, with more significant reductions in HbA1c levels, HOMA-IR, and improvement in dyslipidemic profiles compared to dulaglutide and placebo, leading to an overall enhancement of metabolic health [151]. Tirzepatide is the first-of-class dual GIP-GLP-1 receptor agonist, combining the satiety effects of GLP-1 signaling with the glucagon-induced increase in energy expenditure, potentially resulting in greater weight loss than GLP-1 agonism alone [152]. The Study of Tirzepatide (LY3298176) in Participants With Obesity or Overweight (SURMOUNT-1) (NCT04184622) recently demonstrated the efficacy of once-weekly tirzepatide in adults who are overweight or obese, achieving up to 20.9% reductions in body weight with 15 mg doses [153,154]. Additionally, participants on tirzepatide experienced reductions in blood pressure, fasting plasma glucose, and cholesterol levels [153].

Given these effects, GLP-1 receptor agonists play a critical role in the pharmacological management of the insulin–heart axis, offering benefits that extend beyond glucose control to include cardiovascular protection, weight reduction, and modulation of HF risk. As research continues, these agents are likely to become an increasingly important component of comprehensive cardiovascular care in patients with insulin resistance and heart disease [155].

## 6. DDP4 Inhibitors

Dipeptidyl peptidase 4 (DPP4) is an enzyme expressed in various tissues throughout the human body, including the cardiovascular system, and plays a crucial role in glucose metabolism. DPP4 inhibitors, a newer class of antihyperglycemic drugs, are primarily used to treat patients with diabetes [156]. Five large clinical trials (EXAMINE, SAVOR-TIMI 53, TECOS, CARMELINA, and CAROLINA) have demonstrated the cardiovascular safety of DPP4 inhibitors, although they did not show significant benefits on major cardiovascular adverse events (MACEs) [157,158]. Studies have revealed that DPP4 inhibitors offer protective effects against various cardiovascular diseases, such as hypertension, valvular sclerosis and calcification (particularly in the aortic valve), coronary atherosclerosis, and HF [159]. The DPP4 inhibitors primarily work by increasing the concentration of GLP-1 and GIP in pancreatic β-cells, which promotes insulin secretion and effectively controls blood glucose levels [160]. Unlike other hypoglycemic drugs, DPP4 inhibitors manage blood glucose without causing weight gain [161]. Dyslipidemia, particularly elevated triglycerides, increased low-density lipoprotein (LDL), and decreased high-density lipoprotein (HDL), is a significant risk factor for cardiovascular diseases such as coronary atherosclerosis and calcified aortic valve disease. Notably, several studies have shown that DPP4 inhibitors can regulate lipid metabolism, reducing levels of triglycerides, LDL, and free fatty acids [162,163]. Limited research indicates that DPP4 inhibitors affect the expression of hepatic enzymes responsible for lipid oxidation and biosynthesis by modulating the GLP-1 receptor signaling pathway. This leads to decreased intestinal lipid synthesis and secretion and inhibits lipid absorption [164]. Additionally, DPP4 inhibitors increase plasma norepinephrine levels by activating the sympathetic nervous system, which in turn accelerates the mobilization and oxidation of postprandial lipids [165]. Moreover, European guidelines on cardiovascular disease prevention recognize stress as a clinically significant risk factor for patients with high overall cardiovascular risk or diagnosed cardiovascular disease [165]. Given the complex interplay between insulin resistance, dyslipidemia, and cardiovascular health, DPP4 inhibitors offer a multifaceted approach to managing these conditions within the insulin–heart axis [20].

Recent research has expanded our understanding of the mechanisms through which DPP4 inhibitors confer cardiovascular benefits. Beyond their glucose-lowering effects, these agents modulate inflammatory pathways and endothelial function, both of which are critical in the pathogenesis of cardiovascular diseases. DPP4 inhibitors have been shown to reduce oxidative stress and inflammatory markers, contributing to their protective cardiovascular effects [166,167]. Future research should focus on large-scale, long-term studies to further elucidate the cardiovascular benefits of DPP4 inhibitors, especially in populations with high cardiovascular risk [168]. Combining DPP4 inhibitors with other cardiovascular protective agents, such as SGLT2 inhibitors or GLP-1 receptor agonists, may offer synergistic benefits and should be explored in future clinical trials [169,170]. Additionally, the development of novel DPP4 inhibitors with enhanced selectivity and potency may improve therapeutic outcomes. Understanding patient-specific factors that predict response to DPP4 inhibitor therapy could also lead to more personalized and effective treatment strategies [171].

## 7. Thiazolidinediones

Thiazolidinediones (TZDs), including rosiglitazone, pioglitazone, and the now withdrawn troglitazone, are oral antidiabetic drugs that have demonstrated benefits in conditions associated with insulin resistance (IR). These agents function by activating peroxisome proliferator-activated receptor-gamma (PPAR-γ), a nuclear receptor that influences the transcription of various genes, including those encoding glucose transporter type 4 (GLUT4) receptors, lipoprotein lipase (LPL), and other enzymes critical to energy homeostasis. Through PPAR-γ activation, TZDs reduce insulin resistance in adipose tissue, muscle cells, and the liver [172]. The high abundance of PPAR-γ in adipocytes suggests that these cells might engage in endocrine signaling with skeletal muscles and hepatocytes, potentially mediated by molecules such as free fatty acids (FFAs) and TNF-α. By improving dyslipidemia and reducing IR, TZDs help to lower cardiovascular disease (CVD) risk. Pioglitazone, in particular, has been shown to reduce the incidence of myocardial infarction (MI) and ischemic strokes. This has led to a more nuanced understanding among clinicians of how to balance the benefits and risks of TZD therapy, allowing for better patient selection for these drugs [173]. Moreover, TZDs have been demonstrated to delay beta-cell dysfunction, as evidenced by specific indices, by protecting against oxidative stress and preserving the integrity of pancreatic islets [174,175].

Within the context of the insulin–heart axis, TZDs provide a valuable therapeutic option by addressing key metabolic derangements that contribute to cardiovascular risk. The ability of TZDs to improve insulin sensitivity not only aids in glycemic control but also reduces the metabolic stress placed on the cardiovascular system, which is crucial in preventing the progression of atherosclerosis and other cardiovascular complications [32,176,177,178,179,180,181,182,183]. Additionally, their role in modulating lipid profiles—by lowering triglycerides and potentially raising high-density lipoprotein (HDL) cholesterol—further enhances their cardioprotective effects [179].

However, the use of TZDs has been complicated by concerns over adverse effects, particularly with rosiglitazone, which has been associated with an increased risk of HF [180]. This highlights the need for careful patient selection and monitoring when using these agents. Pioglitazone, on the other hand, has been more favorably viewed due to its broader cardiovascular benefits, though it still requires judicious use due to risks such as weight gain, fluid retention, and bone fractures [181,182].

Recent studies suggest that beyond their established mechanisms, TZDs may exert additional protective effects on the cardiovascular system by reducing inflammation and oxidative stress, both of which are central to the pathogenesis of cardiovascular diseases. Furthermore, TZDs may influence endothelial function, thereby improving vascular health and reducing the risk of adverse cardiovascular events [183].

## 8. Insulin Resistance and Heart Failure

Insulin resistance and metabolic disorders are prevalent among patients with HF, irrespective of the presence of diabetes [184]. In individuals with T2DM, HF can be precipitated via systemic, myocardial, and cellular pathways [184]. Hyperglycemia and hyperinsulinemia promote atherosclerosis by stimulating the proliferation of vascular smooth muscle cells and inducing inflammation [39]. Notably, the prediabetic condition is associated with a significantly increased risk of adverse outcomes in patients with HFrEF. Moreover, insulin resistance is common even in non-diabetic HF patients and has been linked to poor prognosis [185]. The mechanisms that relate insulin resistance to unfavorable clinical outcomes in these patients are not fully understood. However, evidence suggests that in HF patients, reduced insulin sensitivity correlates with higher mortality, independent of body composition and other established risk factors, and may influence the pathophysiology of HF progression [185]. Therefore, targeting reduced insulin sensitivity could potentially benefit patients with HF.

### 8.1. The Role of Sacubitril/Valsartan

Entresto (sacubitril/valsartan), a novel angiotensin receptor–neprilysin inhibitor (ARNI), has shown promising effects in this context. A post hoc analysis of the PARADIGM-HF clinical trial revealed that, in patients with T2DM, those treated with sacubitril/valsartan exhibited a more significant reduction in hemoglobin A1c (HbA1c) levels compared to those treated with enalapril [186]. Furthermore, a study by Cosima Chlor et al. demonstrated that sacubitril/valsartan improved both insulin resistance and metabolic profiles in non-obese prediabetic patients with HFrEF [187]. These benefits may stem from the dual effects of RAAS inhibition on both peripheral IR and insulin secretion. Angiotensin II and aldosterone are known to induce IR by disrupting insulin signaling and reducing glucose transport at the cellular level, while also contributing to inflammation, oxidative stress, and pancreatic β-cell apoptosis [188]. Several clinical studies have confirmed that RAAS inhibition can reduce the incidence of diabetes in patients with HF and/or those at risk of coronary artery disease by enhancing insulin sensitivity and secretion [189,190]. RAAS inhibitors, such as ACE inhibitors and angiotensin receptor blockers (ARBs), can help preserve β-cell function, potentially reducing the risk of diabetes [191]. For instance, valsartan has been shown to improve insulin resistance and enhance glucose-stimulated insulin secretion in patients with impaired fasting glucose or impaired glucose tolerance [192]. Additionally, neprilysin inhibition offers promising potential for improving glycemic control, IR, and metabolic outcomes through several mechanisms. Neprilysin is an enzyme responsible for degrading various vasoactive peptides, including biologically active natriuretic peptides (NPs), angiotensins I and II, bradykinin, adrenomedullin, and GLP-1 [193]. Inhibition of neprilysin results in increased levels of NPs, which play a crucial role in metabolism and insulin resistance. Elevated NP levels may enhance lipid mobilization and postprandial oxidation, leading to increased postprandial energy expenditure [194], improve adiponectin synthesis in adipose tissue, thereby improving both insulin resistance and glucose metabolism [195], and promote the browning of adipose tissue, all of which contribute to improved insulin sensitivity [196]. Moreover, increased NP levels can inhibit proinflammatory cytokines, such as interleukin 6 [197]. Additionally, neprilysin inhibition alters levels of other substances, such as increasing bradykinin and GLP-1 while decreasing endothelin-1, further contributing to improved metabolic control. Given these findings, therapeutically targeting reduced insulin resistance and the prediabetic condition could offer substantial benefits for patients with HF, particularly through agents like sacubitril/valsartan.

### 8.2. The Role of Ventricular Assist Devices (VADs)

The optimal treatment strategy for patients with T2DM and HF remains a topic of ongoing research [198]. While metabolic interventions such as SGLT2 inhibitors have shown promise in improving glucose metabolism, ventricular assist devices (VADs) have also emerged as a vital treatment option for patients with advanced HF. VADs serve both as a targeted therapy and as a bridge to transplantation by assisting cardiac circulation. [199]. Recent studies suggest that VADs may also have a role in glycemic control, reducing the need for antidiabetic drugs and significantly improving HbA1c, insulin requirements, and glucose levels [200,201]. HF is characterized by elevated levels of growth hormone (GH), indicating GH resistance, along with reduced levels of insulin-like growth factor-1 (IGF-1) and IGF-binding protein-3 (IGFBP-3). The elevated GH/IGF-1 ratio is a marker of GH resistance, which is associated with inflammation and impaired hepatic GH signaling mediated by tumor necrosis factor-alpha and interleukin 6 [202]. The placement of a left ventricular assist device (LVAD) in patients with HF has been shown to result in lower levels of circulating growth hormone (GH). Recent studies indicate that LVAD implantation leads to increased expression of insulin-independent glucose transporter type 4 (GLUT4) in muscle tissues, thereby enhancing glucose transport capacity. Additional changes observed include a 50% reduction in pyruvate dehydrogenase kinase-4 (PDK-4) and an increase in differentiation cluster 36 (CD36) and carnitine palmitoyl transferase 1 (CPT-1), both of which are involved in mitochondrial fatty acid uptake. Furthermore, there is an elevation in peroxisome proliferator-activated receptor co-activator 1α (PGC1α) levels in the rectus abdominis muscle of patients [202,203]. The dysfunction of the insulin axis can lead to myocardial dysfunction, resulting in overt HF. At this stage, LVAD implantation plays a critical role in modulating the neuromodulatory effects of insulin on the heart, potentially decelerating, stabilizing, or even reversing the deleterious cascades activated in end-stage HF.

## 9. Future Perspectives

The future of treating the insulin–heart axis holds significant potential, with several promising directions for research and clinical practice. First, further in vitro and in vivo studies are needed to unravel the complex mechanisms leading to insulin resistance, particularly within the context of HF [204,205]. The identification of new biomarkers for early detection of insulin resistance, alongside advances in metabolomics and gut microbiota analysis, could revolutionize personalized medicine approaches [206]. Additionally, exploring lesser-known myokines, hepatokines, miRNA, and adipocytokines as potential therapeutic targets may offer novel strategies for managing insulin resistance [207,208,209,210].

In terms of pharmacological advances, research should focus on developing new agents that target the insulin–heart axis more effectively [211]. This could include the refinement of existing therapies like GLP-1 receptor agonists and SGLT2 inhibitors, as well as the development of combination therapies that address multiple aspects of insulin resistance and HF simultaneously. For example, combining RAAS inhibitors with agents that target neprilysin or other pathways involved in insulin signaling could offer synergistic benefits. Moreover, gene therapy and novel biologics that modulate the expression of key proteins involved in insulin signaling and metabolic regulation represent another frontier in the treatment of insulin resistance and HF [212,213]. As our understanding of the molecular underpinnings of these conditions deepens, these innovative therapies could provide more personalized and effective treatment options for patients [214,215,216]. Ultimately, the integration of these advances into clinical practice will require ongoing collaboration between researchers, clinicians, and regulatory bodies to ensure that new therapies are both safe and effective for the diverse populations affected by insulin resistance and HF.

## 10. Conclusions

IR is a precursor to numerous pathological conditions, including CVD, obesity, inflammation, dyslipidemia, endothelial dysfunction, oxidative stress, and hypertension. Effective management strategies should focus on both primary prevention, such as dietary modifications and physical activity, and pharmacological interventions aimed at improving insulin sensitivity. Several agents, including metformin, GLP-1 receptor agonists, SGLT2 inhibitors, and thiazolidinediones, have shown promise not only in improving metabolic control but also in reducing cardiovascular events. The broader implications of these strategies on patient outcomes are significant, as they hold potential to reduce morbidity and mortality rates, ultimately alleviating the public health burden associated with insulin resistance and CVD. However, many gaps in our understanding remain. For instance, the precise molecular mechanisms through which IR contributes to CVD, particularly at the cellular and myocardial levels, require further exploration. Future research should focus on elucidating these mechanisms to optimize treatment strategies and develop targeted therapies. Additionally, there is a need for large-scale clinical trials to evaluate the long-term effects of current and emerging therapeutic approaches on cardiovascular outcomes. To streamline interventions, therapies targeting insulin resistance can be grouped into two broad categories: lifestyle interventions and pharmacological treatments. Lifestyle changes, such as exercise and dietary interventions, serve as the cornerstone of prevention, while pharmacological agents enhance insulin sensitivity and provide additional cardiovascular benefits. These combined strategies are essential for reducing the long-term complications of insulin resistance and improving patient quality of life.

## Data Availability

No dataset was generated for the publication of this article.
